# Improved salt stress resilience, growth, and quality of soilless basil through biostimulant application

**DOI:** 10.1038/s41598-025-19670-8

**Published:** 2025-10-10

**Authors:** Boran Ikiz, Hayriye Yildiz Dasgan, Sibel Balik, Abdullah Aldiyab, Nazim S. Gruda

**Affiliations:** 1 Department of Horticulture, Faculty of Agriculture, University of Cukurova, 01330 Adana, Turkey; 2 Division of Horticultural Sciences, Institute of Plant Sciences and Resource Conservation, University of Bonn, 53113 Bonn, Germany

**Keywords:** *Ocimum basilicum* L. var. *purpurascens*, Saline water, Hydroponics, Physiological responses, Yield, Antioxidants, Environmental sciences, Physiology, Plant sciences

## Abstract

Salinity is a major abiotic stress that disrupts ion balance, water uptake, and plant metabolism, ultimately reducing growth and productivity. Climate change, induced evaporation, and altered rainfall patterns are accelerating salinization, posing a challenge to soilless systems where water quality directly impacts nutrient availability. Basil, a salt-sensitive and high-value aromatic herb, shows marked physiological decline under salinity, including reduced water and nutrient uptake, impaired photosynthetic activity, disruption of ion balance, and increased oxidative stress. Here, we evaluated the potential of biostimulants—amino acids, arbuscular mycorrhizal fungi (AMF), plant growth-promoting rhizobacteria (PGPR), fulvic acid, chitosan, and vermicompost—to alleviate salt-induced stress in basil grown with 50 mM NaCl in a floating culture system. Salt stress reduced leaf yield by 41.6%, stomatal conductance by 65.7%, and antioxidant enzyme activities. Among the biostimulants, PGPR and vermicompost were the most effective, increasing yield by over 90% compared to salt-stressed plants. These treatments enhanced antioxidant enzyme activities (APX, CAT, GR, SOD), increased phenolics, flavonoids, and vitamin C, and reduced lipid peroxidation (up to 74.3% lower MDA). Moderate improvements were observed with amino acids, AMF, and chitosan, while fulvic acid showed limited effectiveness. Overall, PGPR and vermicompost strengthened basil’s resilience to salinity by reducing oxidative stress and enhancing physiological performance. These findings support their use as sustainable tools in managing saline conditions. Future studies should evaluate the biostimulant effectiveness under higher salinity and poor-quality water, and assess their impact on different basil cultivars, including essential oil and aroma-related traits.

## Introduction

Climate change, primarily driven by fossil fuel combustion and deforestation, intensifies global temperatures and alters precipitation patterns. These changes directly impact greenhouse-based vegetable production, where precise environmental control is crucial for maintaining yield and quality^1,2^. Although elevated CO₂ may enhance plant growth, increased heatwaves, droughts, and humidity fluctuations compromise productivity and quality even under protected cultivation^3,4^. Moreover, changing climate conditions can exacerbate pest and disease pressures in greenhouse environments, necessitating adaptive strategies to maintain sustainable production^5,6^. Among the many adverse effects of climate change, salinization has emerged as a significant constraint, particularly in irrigated systems. Over 20% of irrigated agricultural land is affected by salinity, and projections indicate that nearly 50% of arable land may be impacted by 2050^7^.

Increasing soil salinity is a global and growing issue that poses significant risks to agricultural productivity^8^. It is a fundamental issue in intensive farming systems and semi-arid regions, where precipitation and evapotranspiration are not in balance. However, elevated salinity also impacts soilless growing systems, whether they are open or closed^9^. Particularly in the latter case, where the nutrient solution is reused multiple times within the crop system, salt buildup in the root zone becomes unavoidable. This leads to potential issues with plant functionality and growth, ultimately reducing crop productivity^10,11^. Moreover, in both open and closed soilless cultivation systems, the disposal of spent nutrient solutions into the environment contributes to soil degradation, leads to significant environmental harm, and results in inefficient use of valuable resources.^12^.

Biostimulants are either naturally derived or synthetic compounds that promote plant development, improve yield, and increase resilience to stress by influencing physiological functions, without functioning as standard fertilizers or pesticides^13^. In the agricultural context, biostimulants primarily aim to boost nutrient use efficiency, improve the quality of crops, and alleviate abiotic stress effects, providing a sustainable and innovative strategy to enhance productivity with minimal environmental harm^14^. Their use contributes to lowering the reliance on chemical inputs in both agriculture and plant protection practices^15^. Various groups of biostimulants are utilized in agricultural practices to support plant growth and development^16^. This study assessed the effectiveness of six biostimulants—amino acids, fulvic acid (FA), plant growth-promoting rhizobacteria (PGPR), chitosan, arbuscular mycorrhizal fungi (AMF) and vermicompost—in mitigating salt induced stress in basil cultivated in a floating culture system.

Amino acids, which are among the biostimulants, can be obtained through chemical synthesis, as well as from plant-based proteins such as algae, corn, and soybean, or from animal-derived proteins via chemical or enzymatic hydrolysis.^16–19^. They are regarded as potent biostimulants due to their positive influence on plant growth and development. Numerous studies conducted on a wide range of crops have revealed their diverse advantages. These compounds enhance fertilizer utilization, facilitate more efficient nutrient and water absorption, and improve photosynthetic performance. Together, these physiological benefits lead to increased flowering, better fruit set, and enhanced yield, underscoring their essential role in advancing agricultural efficiency^20^. Humic substances are natural organic compounds formed through the decomposition of plant and animal residues under the influence of microorganisms and geochemical processes; they are classified into humin, humic acid, and fulvic acid based on their solubility at different pH levels. Among these, fulvic acid stands out due to its relatively low molecular weight and functional groups that are rich in oxygen and poor in carbon^21,22^. Fulvic acids support plant development by enhancing photosynthetic activity, stimulating the production of growth hormones, improving nutrient uptake and pH balance, and strengthening resistance against both biotic and abiotic stresses^22^.

PGPR are a group of beneficial bacteria that inhabit the rhizosphere and contribute to plant development. These microorganisms enhance plant growth through both direct and indirect mechanisms, such as improving nutrient availability or suppressing harmful pathogens. Their interaction with plant roots plays a key role in promoting sustainable agricultural practices^23^. PGPR significantly contribute to alleviating the effects of salinity stress by improving the plant’s ability to absorb water, enhancing nutrient uptake efficiency, and promoting the synthesis of compatible solutes such as proline, glutamate, glycine betaine, various sugars, and carbohydrates^24,25^. Chitosan is a naturally occurring biopolymer obtained from chitin, which is present in the exoskeletons of crustaceans like shrimp, crabs, and lobsters. It is formed through the deacetylation process of chitin, producing a polysaccharide with a positive charge^26,27^. Thanks to its biocompatibility, biodegradability, non-toxic nature, and antimicrobial activity, chitosan is applied in agriculture as a biopesticide, biofertilizer, and biostimulant. These applications help promote plant growth, increase crop productivity, and provide protection against abiotic stresses and various plant diseases^28,29^.

AMF are commonly found in soils and form mutualistic relationships with the majority of terrestrial plants^30^. AMF are essential root-associated symbionts that significantly contribute to improving crop growth and assist host plants in developing resilience to abiotic challenges, including drought and salinity^31,32^. Vermicompost is an organic fertilizer formed through the decomposition of organic waste by earthworms, containing nutrient-rich castings that boost soil fertility and promote soil health^33,34^. Vermicompost has been shown to function as both a stimulant for plant growth and a natural defense enhancer for crops^35^. These characteristics enable vermicompost to enhance plant growth and provide natural protection for crops. Additionally, it improves soil texture, increases water-holding capacity, and promotes aeration while slowly supplying nutrients to plants, positioning it as an environmentally friendly and sustainable biostimulant^36^. Vermicompost has proven to be a highly efficient and promising biostimulant, achieving notable success in both conventional soil cultivation and soilless greenhouse vegetable production methods^37^.

The selection of these six biostimulants was based on their complementary mechanisms of action in mitigating salinity stress. Amino acids and fulvic acid enhance nutrient uptake and photosynthetic efficiency; PGPR improve root health, water absorption, and osmolyte production; chitosan stimulates plant defense and growth; AMF enhance nutrient acquisition and salt tolerance through symbiotic interactions; and vermicompost enriches the rhizosphere environment by releasing nutrients and beneficial compounds directly into the nutrient solution. Together, they represent diverse and synergistic strategies to improve basil growth and resilience under saline conditions.

Basil (*Ocimum basilicum* L.), a member of the Lamiaceae family, is well known for its rich aromatic qualities and serves multiple purposes as a culinary herb, medicinal plant, and ornamental species^38^.The selection of suitable plant species plays a critical role in soilless agricultural systems, and basil stands out in this context as an annual herb of considerable importance. Its fresh and dried leaves are widely used in culinary applications^39^, while its diuretic and stimulant properties have led to its recognition as a medicinal plant^40^, and it is also utilized in the perfume industry^41^. This diverse range of applications has made basil a commonly selected species in hydroponic and aquaponic systems^42–44^. Moreover, various studies have demonstrated that basil exhibits higher yields under soilless cultivation conditions compared to conventional soil based systems^45^.Salinity is a major limiting factor in basil cultivation, as salt stress triggers various morphophysiological disruptions, including nutrient imbalances, decreased stomatal conductance, and reduced rates of transpiration and photosynthesis. These effects collectively lead to stunted growth and lower biomass production in the plant^46^.

Climate change induced salinization makes soilless basil cultivation particularly vulnerable. Unlike soil based systems, soilless culture lacks natural buffering capacity, causing salts to accumulate rapidly in the root zone and intensifying physiological stress. This challenge becomes more pronounced in greenhouses when saline or brackish water is used for irrigation. In this study, we applied six biostimulants with different modes of action, including enhancing nutrient uptake, strengthening antioxidant defenses, and promoting beneficial plant microbe interactions. We hypothesized that these treatments would improve basil growth and tolerance to salt stress in floating hydroponic culture by increasing antioxidant enzyme activity, reducing oxidative damage, and maintaining ion balance. By providing mechanistic insights and practical solutions, this work aims to support sustainable basil production in floating culture systems and promote the use of marginal water resources, such as brackish water, in greenhouse environments where soil buffering is absent.

## Materials and methods

### Plant material and growing conditions

**Fig. 1:**
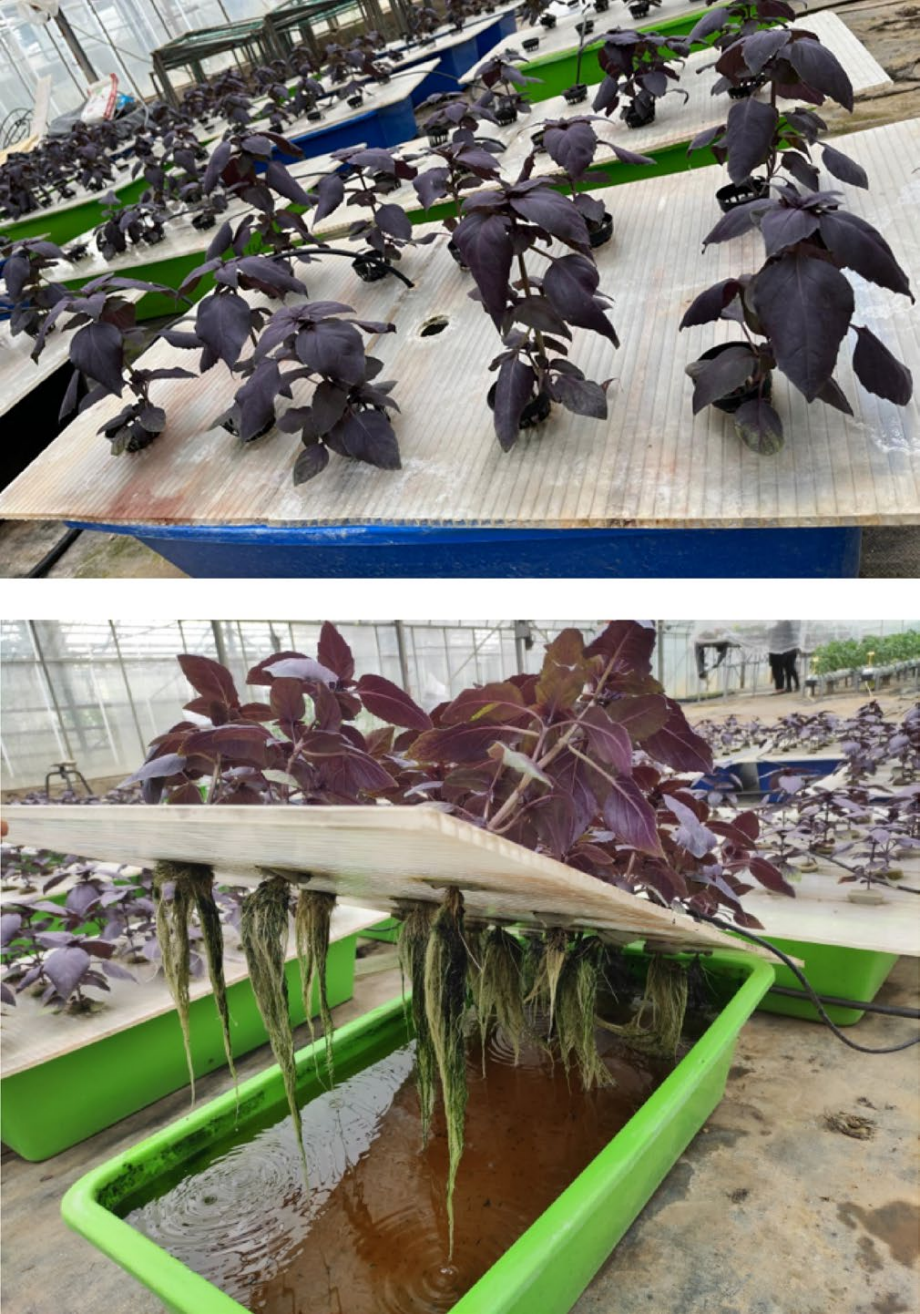
Basil plants were grown hydroponically under saline water and treated with various biostimulants.

The research was conducted in a greenhouse located at 36°59′N, 35°18′E, at an altitude of 20 m above sea level during the spring season of 2023. During daylight hours, temperatures within the greenhouse varied from 24 ± 2 °C, whereas at night, they ranged from 17 + 2 °C. The relative humidity persisted at 60–70%, and the plants were exposed to natural sunlight. Basil particularly the ‘Rosie’® cultivar with purple-red color from Enza Zaden seed company, was utilized as the plant material. A hydroponic system utilizing 50-L culture pots was established, with plant roots immersed in aerated nutrient solution. The experiment employed a randomized complete block design, consisting of four replicates per treatment and ten plants per replicate, with each tank representing a single repeat (Fig. 1).

The spacing between the rows of basil plants was 15 × 15 cm, resulting in a plant density of 44.44 plants per square meter. A nutrient solution consisting of nitrogen (N) 160 to 200, phosphorus (P) 30–40, potassium (K) 220–250, calcium (Ca) 140–160, magnesium (Mg) 40–50, Iron (Fe) 2.5–5.0, manganese (Mn) 0.25–0.4, Boron (B) 0.25–0.40, Zinc (Zn) 0.20–0.50, copper (Cu) 0.02–0.05 and molybdenum (Mo) 0.04–0.07 was used to develop the basil plants in the control treatment (in mg L^‒1^)^47^. A 50 mM NaCl salt treatment was applied to the hydroponically cultivated basil plants. In a 50 mM NaCl salinized nutritional solution, the biostimulants of amino acids, chitosan, fulvic acid, PGPR, and vermicompost were added. Basil seeds were sown on March 22, 2023. Basil seedlings were transplanted to the hydroponic system on April 7, 2023. The basil experiment concluded on May 31, 2023. The pH of the nutrient solution was carefully maintained between 5.5 and 6.0, while the electrical conductivity (EC) was kept at 2.0 dS m^−1^ in the control treatment throughout the plant growth period. Seven days after the transplantation of basil seedlings into the hydroponic system, a salinity treatment of 50 mM NaCl was initiated. Concurrently, biostimulant applications were started to investigate their potential in mitigating salt-induced stress. The nutrient solution was replaced weekly, and the biostimulants were added each time to the newly prepared solution. Only AMF was applied once to the root zone during seed sowing 1000 spors per seedling, while the seedlings were being raised.

## Biostimulants

Amino acid and fulvic acid, products of the Köklü Group, were employed under the trade designations “Aminoset”® and “Sacaka WS”®, respectively. Aminoacid comprises 50% organic matter, 20% organic carbon, 4% organic nitrogen, and 30% free amino acids. Conversely, fulvic acid comprises 80% organic matter and 70% fulvic acid. The concentrations of amino acid and fulvic acid in the root media of basil were established at 100 mg/L and 40 mg/L, respectively^34^. Furthermore, “ERS”® (Bioglobal Inc. Co.), a mycorrhizal blend comprising *Glomus intraradices, Glomus aggregatum, Glomus mosseae, Glomus clarum, Glomus monosporus, Glomus deserticola, Glomus brasilianum, Glomus etunicatum*, and *Gigaspora margarita* at a concentration of 1 × 10^4^ g^−1^, was administered to the seeds before sowing at a dosage of 1000 spores per seed^47^.Additionally, “Rhizofill” ® (NG-Biyoteknoloji Ltd. Co.), including *Bacillus subtilis* (1 × 10^9^ ml^−1^), *Bacillus megaterium* (1 × 10^9^ ml^−1^), and *Pseudomonas fluorescens* (1 × 10^10^ ml^−1^), was employed as a PGPR biostimulant at a concentration of 1.0 ml L^−1^^47^. “Adaga”® from Adaga firm, which contains 2.5% N-Acetyl-D-Glucosamine, was utilized as chitosan at a concentration of 300 µl/L in a hydroponic cultivation system. Finally, “EkosolFarm”® (100% organic liquid vermicompost) from Ekosol Tarim company was used as vermicompost at a 2 ml/L concentration^34^.

### Basil leaf harvests

Basil plants were harvested four times during the cultivation period. The first harvest took place five weeks after transplanting, with subsequent harvests at 7–10 day intervals. Removal of the apical part, including the growing tip, stimulated leaf emergence from lateral branches, allowing repeated harvests. The total yield was calculated and expressed in kilograms per square meter (kg m^−2^).

### Plant growth parameters

The height of the basil was measured with a ruler. The color values of the gathered basil leaves were digitally presented using a portable handheld color spectrophotometer (HunterLab, Virginia, USA). The fresh weight (FW) of basil leaves was recorded, subsequently dried at 65 °C for 24 h, and reweighed (DW) to determine the percentage of dry matter content^47^.

(DW = 100 × DW/FW)(38).

### Basil antioxidant measurements

The methodology given by Spanos and Wrolstad^48^ quantified total phenolic content with a modification. The total extracted phenolics were quantified in milligrams of Gallic acid (GA) equivalents, based on absorbance measurements at 765 nm, using a UV visible spectrophotometer (UV-1700 Pharma Spec Shimadzu, Japan). The total flavonoid content in basil leaf samples was quantified using a UV–visible spectrophotometer (UV-1700 Pharma Spec Shimadzu, Japan) at 765 nm, as described by Quettier et al.^49^. The total flavonoid compounds were quantified using a calibration established with standards. The measurement of Vitamin C was performed using the modified method described by Elgailani et al.^50^. Basil leaves were homogenized with a high-speed blender, and 5 mL of the basil extract was then mixed with 45 mL of 0.4% oxalic acid before filtering. The filtrate, consisting of 1 mL of extract and 9 mL of 2,6-dichlorophenolindophenol sodium salt, was analyzed for transmittance at 520 nm with a UV spectrophotometer.

### Mineral elements, sodium, and nitrate analysis

The levels of potassium (K), magnesium (Mg), calcium (Ca), iron (Fe), manganese (Mn), and zinc (Zn) in basil leaves were evaluated using an atomic absorption spectrophotometer. Three individual plants from each replication were dried at 65 °C for 48 h and ground using a 20-mesh sieve mill. The generated leaf powder was incinerated in a furnace at 550 °C for 8 h, and the resultant ash was solubilized in 3.3% hydrochloric acid (HCl). Fe, Mn, Zn, and Cu concentrations were quantified using atomic absorption spectrometry in absorbance mode. Conversely, the K, Ca, Mg, and Na concentrations were assessed in emission mode^51^. The Kjeldahl and Barton methods assessed leaf nitrogen and phosphorus concentrations^52^. The colorimetric assessment of nitrate accumulation in basil leaves was performed via the transnitration of salicylic acid, as outlined by Cataldo et al.^53^ and adapted by Dasgan et al.^52^.

### Activities of antioxidative enzymes

The activity of antioxidant enzymes was evaluated by extracting enzymes from 0.5 g of basil leaf tissue using a mortar and pestle, along with 5 mL of extraction solution composed of 50 mM potassium-phosphate buffer at pH 7.6 and 0.1 mM disodium ethylenediaminetetraacetate. After centrifugation of the homogenate for 15 min at 15,000 g, the supernatant fraction was employed for enzyme tests. All enzyme extraction protocols were performed at 4 °C, and activity was assessed following references^14,54,55^. The activity of SOD was evaluated by observing the reduction of nitro blue tetrazolium (NBT) caused by superoxide radicals at a wavelength of 560 nm. A unit of SOD activity is defined as the quantity of enzyme necessary to prevent 50% of NBT degradation by photochemical methods. The catalase (CAT) activity was assessed by observing the breakdown rate of hydrogen peroxide (H_2_O_2_) at 240 nm. For this analysis, a 50 mM phosphate buffer at pH 7.6, supplemented with 0.1 mM EDTA, 0.1 ml of 100 mM H_2_O_2_, and enzyme extract, was included in the reaction medium, achieving a final volume of 1 ml. The activity of APX was assessed by quantifying ascorbate consumption at 290 nm. An APX activity unit is the enzyme quantity necessary to metabolize one mole of ascorbate per minute. The activity of GR was assessed by measuring the absorbance of nicotinamide adenine dinucleotide phosphate (NADPH) at 340 nm and its oxidation rate in the presence of the enzyme. The enzyme volume necessary to oxidize 1 mol of NADPH per minute was designated as 1 unit of GR activity.

### Determination of MDA (malondialdehyde) and proline

Lipid peroxidation level was calculated based on the MDA (malondialdehyde) level determined using the thiobarbituric acid (TBA) reaction method^14,56^. Absorbance was measured at 532 nm after centrifugation of the supernatant at 10.000 g for 10 min at 4 °C. Any non-specific absorption at 600 nm was subtracted from the values obtained. The proline content was quantified from aliquots of leaf crude extracts using the method outlined by Magne and Larher^57^ improved by Dasgan et al.^58^.

### Statistical analysis

The effects of the treatments on morphological, physiological, and biochemical traits, together with enzyme activities, were evaluated using the JMP statistical tool (Version 7.0, Statistical Software, 2007). Differences between treatment means were assessed using the least significant difference (LSD) test at the 5% significance level. In addition, all the independent variables were subjected to principal component analysis (PCA) and multiple variable analyses by Pearson correlation matrix ClustVis software (https://biit.cs.ut.ee/clustvis/; accessed on 21 June 2025).

This study complies with relevant institutional, national, and international guidelines and legislation.

## Results

### Yield, growth, and color parameters of hydroponic basil

**Fig. 2:**
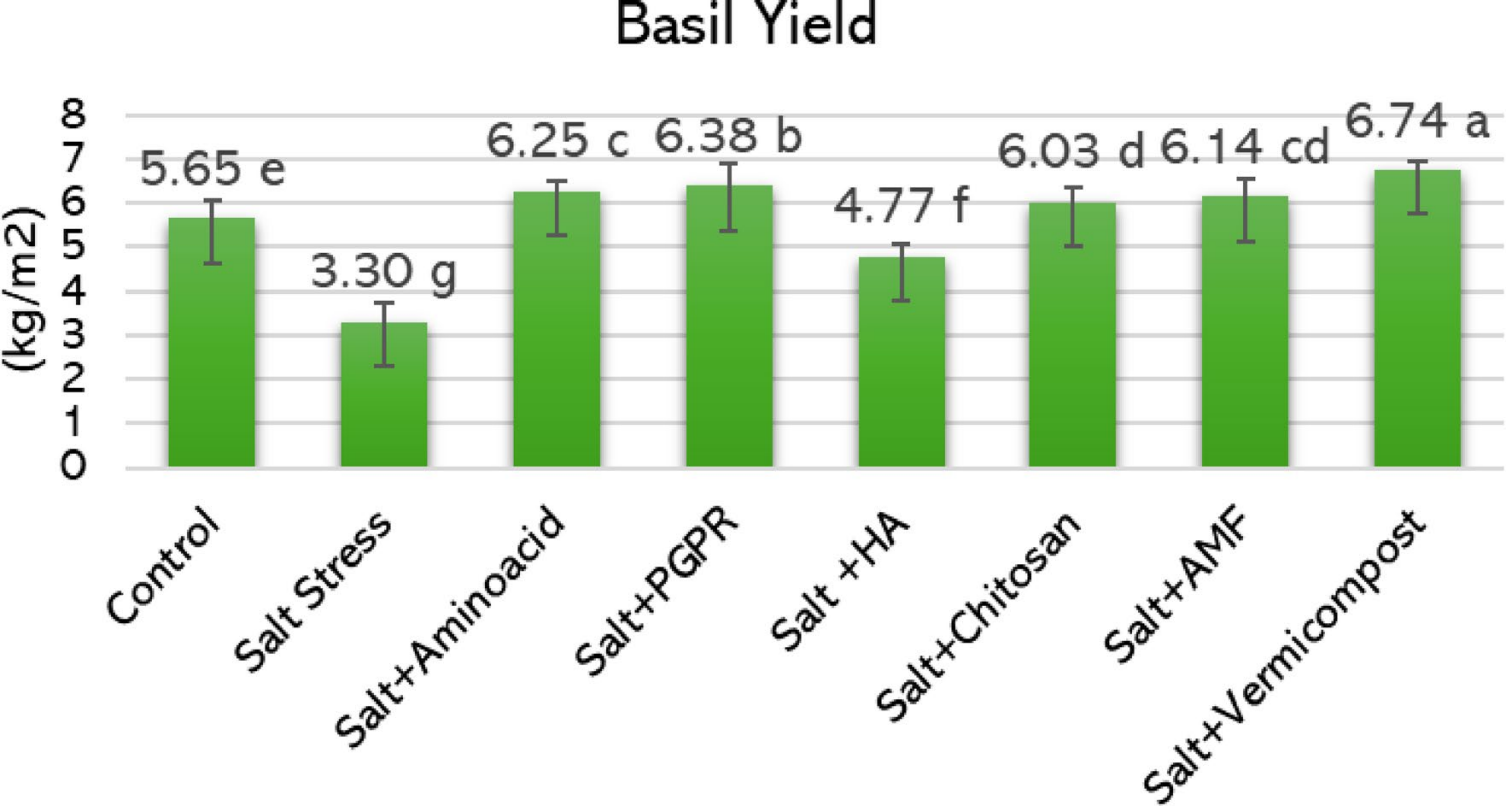
Effects of biostimulant applications on the yield of hydroponically grown basil under 50 mM salinity stress. Different letters on the histogram bars indicate statistically significant differences according to the LSD test (*p* < 0.05). PGPR, plant growth-promoting rhizobacteria; FA, fulvic acid; AMF, arbuscular mycorrhizal fungi.

As shown in Fig. 2, salt stress significantly reduced basil leaf yield from 5.65 kg m^−2^ under non-saline conditions to 3.30 kg m^−2^ under 50 mM NaCl, representing a 41.6% decline. However, all biostimulant applications improved yield compared to the salt-stressed group. Among the treatments, vermicompost led to the highest yield (6.74 kg m^−2^), corresponding to a 104.2% increase over salt stress and a 19.3% improvement over the control. PGPR application followed closely, resulting in 6.38 kg m^−2^ a 93.3% increase relative to salt and 12.9% higher than the control. Amino acid treatment produced a 6.25 kg m^−2^ yield, which was 89.4% greater than salt stress and 10.6% higher than the control. AMF and chitosan also conferred substantial improvements, with respective yields of 6.14 and 6.03 kg m^−2^, corresponding to 86.1% and 82.7% increases compared to salt-stressed plants. In contrast, the yield observed with fulvic acid application was 4.77 kg m^−2^ representing a 44.5% increase over salt stress, yet 15.6% lower than the control.

The mean plant height in the control group was 29.33 cm, whereas exposure to 50 mM NaCl significantly reduced plant height to 15.33 cm, representing an approximate 48% decrease (Table 1). Among the biostimulant treatments, the highest plant height (41.33 cm) was recorded with PGPR application, corresponding to a 170% increase over the salt treatment and a 41% improvement compared to the control. Vermicompost-treated plants reached a similar height (39.66 cm) and were statistically grouped with PGPR, indicating comparable efficacy under saline conditions. Chitosan and AMF treatments also significantly improved plant height, reaching 37.33 and 36.66 cm, respectively, surpassing the control by approximately 25–27%. Amino acid application resulted in a height of 33.33 cm, 117% higher than the salt treatment and slightly (14%) greater than the control. The combined application of humic and fulvic acid yielded a plant height of 25.66 cm, reflecting a 67% increase compared to the salt treatment but remaining approximately 14% lower than the control. The shortest plants (15.33 cm) were observed in the salt group, significantly lower than all other treatments.

**Table 1:** Effects of biostimulant applications on plant growth parameters of hydroponically grown basil under 50 mM NaCl salinity.

Treatment	Plant height (cm)	Dry matter (%)	HUE
Control	29.33 de	6.97 e	307.65 d
Salt	15.33 f	9.84 d	310.03 cd
Salt + amino acid	33.33 cd	10.90 c	313.25 b
Salt + PGPR	41.33 a	11.77 b	319.72 a
Salt + FA	25.66 e	9.47 d	307.82 d
Salt + chitosan	37.33 ac	9.51 d	312.89 b
Salt + AMF	36.66 bc	10.84 c	311.89 bc
Salt + vermicompost	39.66 ab	12.93 a	318.60 a
LSD_0.05_	4.21	0.8	2.64
*P*	0.0001	0.0001	0.0001

Values followed by different letters within the same column are significantly different according to the LSD test (*p* < 0.05). NS, non-significant; PGPR, plant growth-promoting rhizobacteria; FA, fulvic acid; AMF, arbuscular mycorrhizal fungi.

The application of PGPR resulted in the highest dry matter content (11.78%), reflecting a 20% increase relative to the control (6.97%) and a 20% improvement over the salt treatment (9.84%) (Table 1). Treatments with amino acids (10.91%) and AMF (10.85%) also enhanced dry matter accumulation, representing increases of approximately 11% and 10%, respectively, compared to the salt treatment, and approaching values near or slightly above the control. In contrast, chitosan (9.51%) and fulvic acid (9.47%) showed only minimal increases in dry matter content, about 4% and 3%, respectively, compared to the salt treatment. These values remained substantially lower than those observed in the control group.

The application of PGPR was the most effective in preserving leaf color, resulting in a HUE value of 319.7, approximately 3% higher than the salt treatment (310.0) and about 4% greater than the control (307.7). Vermicompost yielded a similarly high HUE value of 318.6. It was statistically grouped with PGPR in the highest category (“a”), indicating that these two treatments most effectively preserved the characteristic purple pigmentation of basil leaves under salinity stress. Since the study was conducted on purple basil, the HUE values reflect variations in the intensity and retention of purplish coloration. Amino acid (313.3) and chitosan (312.9) applications slightly increased HUE values compared to the salt treatment, with improvements of approximately 1.1% and 0.9%, respectively. The HUE value recorded in the AMF treatment was 311.9, nearly identical to that of the salt treatment. In contrast, the application of fulvic acid resulted in a HUE value of 307.8, equivalent to the control, indicating no substantial improvement in leaf pigmentation under saline conditions.

### Nitrate and antioxidant bioactive compounds in basil

The nitrate concentration in the leaves of control plants was 211.9 mg kg^−1^ DW (Table 2). Salt stress reduced nitrate accumulation to 180.0 mg kg^−1^ DW, corresponding to an 18% decrease. Treatments with amino acids, AMF, vermicompost, and fulvic acid increased nitrate concentrations compared to the salt treatment, approaching levels similar to the control. In contrast, chitosan and PGPR treatments resulted in lower nitrate values (187.7 and 186.5 mg kg^−1^ DW, respectively), about 4% higher than the salt treatment but 12–13% lower than the control.

**Table 2:** Effects of biostimulants on nitrate content and antioxidant compounds in hydroponically grown basil exposed to 50 mM NaCl salinity.

Treatment	Nitrate (mg kg DW^−1^)	Vitamin C (mg100g FW^−1^)	Total phenols (mg GA 100 g FW^−1^)	Total flavonoids (mg RU 100 g FW^−1^)
Control	211.93 a	17.10 d	32.06 g	13.88 f.
Salt	180.00 b	19.13 c	39.46 ef	17.59 e
Salt + amino acid	212.76 a	22.16 ab	46.56 c	22.03 bc
Salt + PGPR	186.53 b	22.90 a	52.90 b	23.10 ab
Salt + FA	203.83 a	18.63 cd	37.67 f.	19.36 de
Salt + chitosan	187.66 b	21.10 b	43.36 cd	20.31 cd
Salt + AMF	209.33 a	19.43 c	41.16 de	19.91 cd
Salt + vermicompost	208.9 a	23.06 a	57.60 a	24.19 a
LSD_0.05_	9.13	1.53	3.2	2.16
*P*	0.0001	0.0001	0.0001	0.0001

Values followed by different letters within the same column are significantly different according to the LSD test (*p* < 0.05). NS, non-significant; PGPR, plant growth-promoting rhizobacteria; FA, fulvic acid; AMF, arbuscular mycorrhizal fungi.

Vitamin C content in the control was 17.10 mg 100 g^−1^ FW, while salt stress increased it to 19.13 mg 100 g^−1^ FW, corresponding to a 12% rise. Biostimulant treatments further enhanced vitamin C accumulation under saline conditions. The highest values were observed in the vermicompost (23.06 mg 100 g^−1^) and PGPR (22.90 mg 100 g^−1^) treatments, representing approximately 20% increases compared to salt and 35% and 34% increases relative to the control, respectively. The amino acid treatment resulted in 22.16 mg 100 g^−1^, indicating a 16% increase over salt and nearly 30% over the control. Chitosan yielded 21.10 mg 100 g^−1^, corresponding to a 10% increase compared to salt and a 23% increase relative to the control. The AMF application produced 19.43 mg 100 g^−1^, a value close to the salt treatment’s and 14% higher than the control. In contrast, fulvic acid resulted in 18.63 mg 100 g^−1^, slightly lower than the salt treatment (− 3%) but about 9% higher than the control.

Salt stress increased total phenolic content in basil leaves from 32.06 mg GAE 100 g^−1^ FW in the control to 39.46 mg 100 g^−1^ FW, corresponding to a 23% increase (Table 2). All biostimulant treatments, except vermicompost and fulvic acid, enhanced phenolic accumulation under saline conditions. The highest total phenol content was recorded in the PGPR treatment (52.90 mg 100 g^−1^ FW), representing increases of approximately 34% compared to the salt treatment and 65% relative to the control. This treatment was statistically classified in the top group. The amino acid application yielded 46.56 mg 100 g^−1^ FW, corresponding to increases of 18% over salt and 45% over control. Chitosan resulted in a phenol content of 43.36 mg 100 g^−1^ FW (+ 10% vs. salt; + 35% vs. control), while AMF reached 41.16 mg 100 g^−1^ FW (+ 4% vs. salt; + 28% vs. control). These four treatments (PGPR, amino acid, chitosan, and AMF) formed a statistically distinct group with elevated phenol levels. In contrast, vermicompost (37.60 mg 100 g^−1^) and fulvic acid (37.67 mg 100 g^−1^) showed phenol contents slightly lower than the salt treatment (about 4% decrease), though still approximately 17% higher than the control. The lowest phenolic content was observed in the control group.

Total flavonoid content in basil increased significantly under salt stress, from 13.88 mg RE 100 g^−1^ FW in the control to 17.59 mg 100 g^−1^ FW under saline conditions, reflecting a 27% rise (Table 2). Biostimulant treatments further enhanced flavonoid accumulation. The highest flavonoid content was observed in the vermicompost treatment (24.19 mg 100 g^−1^), representing a 37% increase over the salt treatment and a 74% increase relative to the control. PGPR yielded a comparable concentration (23.10 mg 100 g^−1^), corresponding to 31% and 66% increases over salt and control, respectively. Both treatments were statistically grouped in the highest category. The amino acid application resulted in 22.03 mg 100 g^−1^, showing a 25% increase compared to salt and a 59% increase relative to control, and was classified in the second-highest statistical group. Chitosan (20.31 mg 100 g^−1^) and AMF (19.91 mg 100 g^−1^) enhanced flavonoid content by 15% and 13% over salt, and 46% and 43% over control, respectively. These were placed in the intermediate statistical group. The fulvic acid application led to a flavonoid concentration of 19.36 mg 100 g^−1^, approximately 10% higher than salt and 39% above control, and was assigned to a lower statistical group.

### Macro and micro mineral element concentrations of basil

**Fig. 3:**
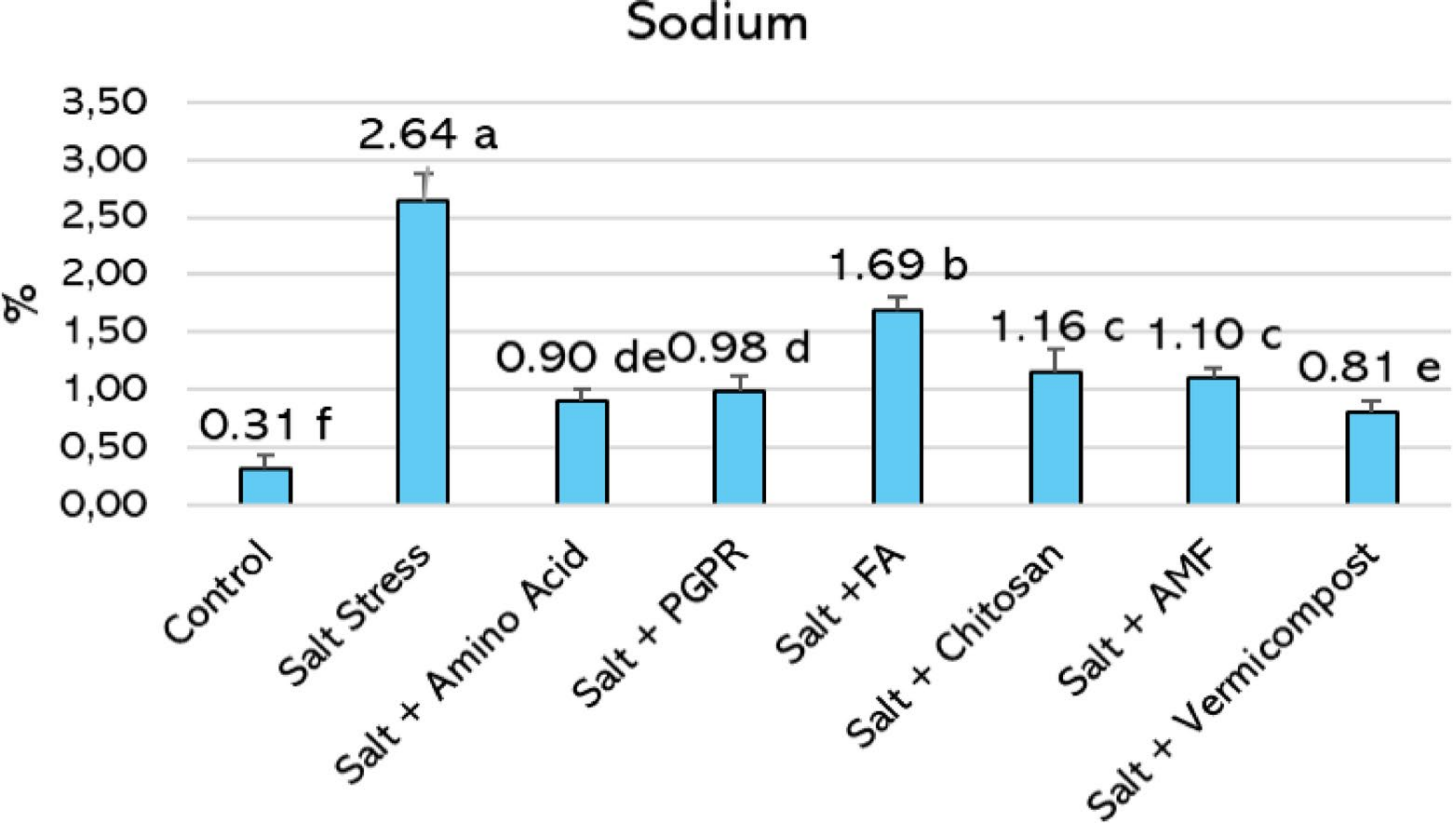
Effects of biostimulant treatments on sodium (Na) content in the leaves of hydroponically grown basil under salt stress. Histograms sharing the same letter are not significantly different (*p* < 0.05).

Saline conditions significantly influence the absorption and distribution of nutritional components in plants. The investigations revealed that basil leaves’ levels of macronutrients (nitrogen, phosphorus, potassium, calcium, and magnesium) considerably decreased under salt stress, whereas sodium accumulation significantly increased (Table 3 and Fig. 3). Salt stress led to a 34% decrease in leaf nitrogen concentration compared to the control. Relative to the salt treatment, nitrogen levels increased by 150% with vermicompost, 142% with PGPR, and 105% with amino acid. Salt stress caused a 41% decline in leaf phosphorus concentration compared to the control. Among the biostimulant treatments, vermicompost led to the highest improvement, increasing phosphorus by 120% relative to salt stress, followed by amino acid (107%) and PGPR (100%). The potassium concentration in the control leaves was 4.05%, but it declined to 2.13% under salt stress, indicating a 47% reduction. Among the treatments, vermicompost led to the highest potassium accumulation, increasing it to 5.98%, corresponding to an increase of approximately 181% compared to the salt treatment. PGPR application also proved highly effective, raising potassium content to 5.84% (+ 174% vs.). Chitosan was the third most effective treatment, increasing potassium to 4.75%, which is 123% higher than the salt treatment. In the control group, the leaf calcium concentration was 1.66%, but it declined to 1.09% under salt stress, reflecting a 34% reduction. PGPR resulted in the highest calcium content among the treatments, increasing it to 2.80%, approximately 157% higher than the salt treatment. Vermicompost also significantly improved calcium levels, raising it to 2.60% (+ 139% vs.), with no statistical difference from PGPR. The third most effective treatment was arbuscular mycorrhizal fungi (AMF), which raised calcium content to 1.91%, representing a 75% increase over the salt-stressed plants. Salt stress resulted in a 41.94% decrease in leaf magnesium concentration compared to the control. Vermicompost application improved significantly, enhancing magnesium levels by 311.11% relative to the salt treatment. PGPR followed closely with a 305.56% increase, while amino acid application elevated magnesium by 255.56% under saline conditions.

**Table 3:** Effects of biostimulants on macro elements in hydroponic basil under salt stress (%).

Treatment	N	P	K	Ca	Mg
Control	5.35 e	0.75 de	4.05 d	1.66 bc	0.31 de
Salt stress	3.53 f	0.44 f	2.13 f	1.09 d	0.18 f
Salt + amino acid	7.23 b	0.91 ab	4.20 cd	1.79 b	0.64 b
Salt + PGPR	8.56 a	0.88 b	5.84 a	2.80 a	0.73 a
Salt + humic acid	5.74 de	0.72 e	3.24 e	1.42 c	0.27 e
Salt + chitosan	6.16 cd	0.81 cd	4.75 b	1.87 b	0.45 c
Salt + AMF	6.52 c	0.83 c	4.63 bc	1.91 b	0.36 d
Salt + vermicompost	8.83 a	0.97 a	5.98 a	2.60 a	0.74 a
LSD_0.05_	0.61	0.067	0.5	0.29	0.068
*P*	0.0001	0.0001	0.0001	0.0001	0.0001

Values followed by different letters within the same column are significantly different according to the LSD test (*p* < 0.05). NS, non-significant; PGPR, plant growth-promoting rhizobacteria; FA, fulvic acid; AMF, arbuscular mycorrhizal fungi.

Sodium concentration increased to 2.64% under salt stress, compared to 0.31% in control conditions (Fig. 3), representing a 751.61% rise. All biostimulant treatments reduced Na accumulation in basil leaves. The most effective were vermicompost, amino acid, and PGPR, which lowered sodium concentrations to 0.81%, 0.90%, and 0.98%, respectively. These values correspond to reductions of 69.32%, 65.91%, and 62.88% relative to the salt treatment.

Salt stress considerably affected the uptake and accumulation of micronutrients in basil leaves, particularly Fe, Mn, Zn and Cu. The application of biostimulants demonstrated notable improvements in the concentrations of these elements under saline conditions (Table 4). In control plants, the Fe concentration was 105.33 ppm (Table 4), whereas salt stress reduced it to 81.66 ppm, reflecting a 22% decline. PGPR and vermicompost treatments elevated Fe levels to 154.00 and 153.00 ppm, respectively, an increase of approximately 88% compared to the salt treatment and around 45% above the control. For manganese, the concentration dropped sharply from 25.33 ppm in the control to 12.00 ppm under salt stress, corresponding to a 53% reduction. Vermicompost and PGPR markedly enhanced Mn content, reaching 53.33 and 52.66 ppm, respectively. These values represent increases of about 344–339% over the salt-stressed plants and 110–108% relative to the control. Zinc levels followed a similar pattern, falling from 6.66 ppm in the control to 3.53 ppm under salt stress, a decrease of 47%. PGPR and vermicompost significantly boosted Zn accumulation, raising concentrations to 14.66 and 14.33 ppm. These represent gains of approximately 315–306% over salt-stressed plants and 120–115% compared to the control. As for copper, control plants contained 5.50 ppm, while salt stress reduced it to 4.40 ppm (− 20%). The application of PGPR and vermicompost effectively improved Cu levels to 10.13 and 10.23 ppm, respectively an increase of about 130% over the salt treatment and 84–86% higher than the control.

**Table 4:** Effects of biostimulants on micro elements in hydroponic basil under salt stress (ppm).

Treatment	Fe	Mn	Zn	Cu
Control	105.33 d	25.33 c	6.66 de	5.50 d
Salt stress	81.66 e	12.00 d	3.53 f	4.40 e
Salt + amino acid	137.66 b	34.66 b	10.33 bc	7.43 c
Salt + PGPR	154.00 a	52.66 a	14.66 a	10.13 a
Salt + humic acid	97.33 d	21.00 c	5.66 e	5.46 d
Salt + chitosan	127.33 bc	42.33 b	8.33 cd	8.33 b
Salt + AMF	124.00 c	35.33 b	10.66 b	7.46 c
Salt + vermicompost	153.00 a	53.33 a	14.33 a	10.23 a
LSD_0.05_	11.54	8.57	2.08	0.57
*P*	0.0001	0.0001	0.0001	0.0001

Values followed by different letters within the same column are significantly different according to the LSD test (*p* < 0.05). NS, non significant; PGPR, plant growth-promoting rhizobacteria; FA, fulvic acid; AMF, arbuscular mycorrhizal fungi.

### Stomatal conductance, RWC, and membrane injury

In control plants, stomatal conductance was recorded at 151.66 mmol m^−2^ s^−1^. Under salt stress, this value dropped sharply to 52.00 mmol m^−2^ s^−1^, representing a 65.7% reduction (Table 5). Among the biostimulant treatments, PGPR showed the most pronounced effect in mitigating the impact of salt stress, elevating stomatal conductance to 130.00 mmol m^−2^ s^−1^ an increase of 150% compared to the salt-stressed group. This value was only 14.3% lower than the control and was statistically grouped with it, suggesting a near-complet recovery. Vermicompost and AMF treatments also significantly enhanced stomatal conductance, reaching 82.66 and 77.00 mmol m^−2^ s^−1^, corresponding to increases of approximately 59% and 48%, respectively, compared to the salt-stressed plants. In control plants, membrane injury was minimal, with an index of only 2.35%. However, under salt stress, this value rose dramatically to 6.07%, representing an increase of approximately 158%, indicating significant damage to cellular membrane integrity. All biostimulant treatments effectively reduced MII compared to the salt-stressed group. The most notable reductions were observed in the PGPR and vermicompost treatments, which lowered the MII to 2.47% and 2.34%, respectively corresponding to reductions of approximately 59% and 61%. These results suggest that both biostimulants substantially alleviated salt-induced membrane damage.

**Table 5:** Effects of biostimulants on stomatal contactance and MII of basil leaf under salt stress.

Treatment	Stomatal conductance (mmol/m^2^/s )	MII (%)
Control	151.66 a	2.35 f
Salt stress	52.00 d	6.07 a
Salt + amino acid	56.66 d	3.22 e
Salt + PGPR	130.00 b	2.47 f
Salt + humic acid	56.66 d	4.55 b
Salt + chitosan	54.66 d	3.81 d
Salt + AMF	77.00 c	4.34 c
Salt + vermicompost	82.66 c	2.34 f
LSD (0.05)	9.98	0.0001
*P*	0.0001	0.19

Values followed by different letters within the same column are significantly different according to the LSD test (*p* < 0.05). NS, non significant; PGPR, plant growth-promoting rhizobacteria; FA, fulvic acid; AMF, arbuscular mycorrhizal fungi; RWC, leaf relative water content; MII, memrane injury index.

### Antioxidative enzyme activities, lipid peroxidation and prolin content

#### Ascorbate peroxidase (APX)

**Fig. 4:**
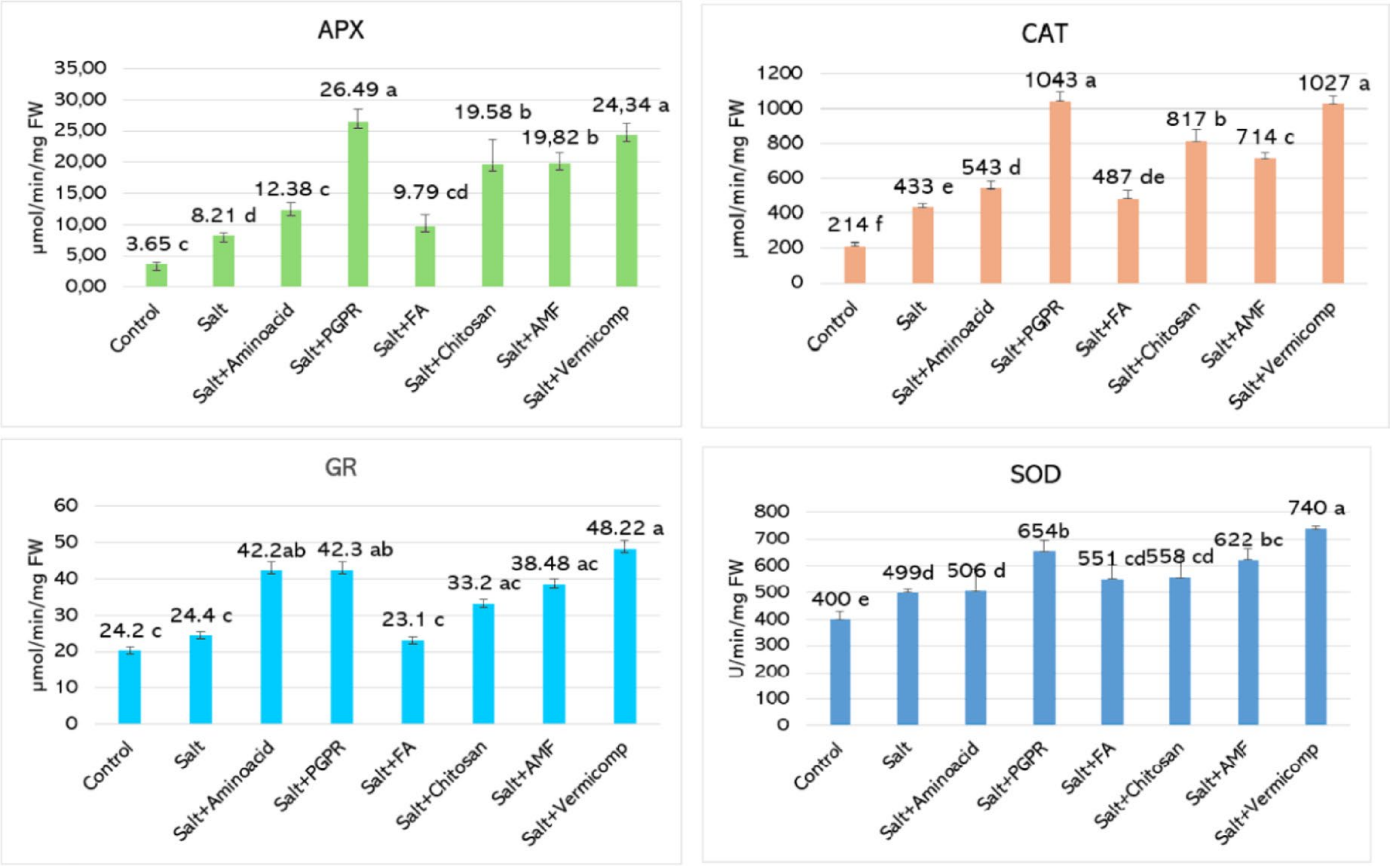
Effects of biostimulants on antioxidant enzyme activities in basil leaves under 50 mM salt stress. Histograms with the same color and letter are not significantly different (*p* < 0.05).

Significant changes were observed in antioxidant enzyme activity under salt stress compared to the control (Fig. 4). In the case of ascorbate peroxidase (APX), enzyme activity was relatively low in control plants, measured at 3.65 µmol min^−1^ mg^−1^ FW. Salt stress markedly increased APX activity to 8.21 µmol min^−1^ mg^−1^ FW, indicating a 125% rise, which reflects an induced antioxidative defense response. Among the biostimulant treatments, PGPR exhibited the most pronounced effect, enhancing APX activity to 26.49 µmol min^−1^ mg^−1^ FW representing a 222.7% increase relative to salt-stressed plants. Vermicompost was also highly effective, raising the enzyme activity to 24.34 µmol min^−1^ mg^−1^ FW, corresponding to a 196.5% increase. Similarly, AMF and chitosan treatments improved APX levels to 19.82 and 19.58 µmol min^−1^ mg^−1^ FW, with increases of 141.4% and 138.5%, respectively. The amino acid application elevated APX activity to 12.38 µmol min^−1^ mg^−1^ FW, a 50.8% improvement compared to salt stress. In contrast, fulvic acid resulted in a modest rise to 9.79 µmol min^−1^ mg^−1^ FW, which corresponds to a 19.2% increase and remained statistically similar to the salt-stressed group.

#### Catalase (CAT)

Biostimulant applications markedly enhanced catalase (CAT) activity under salt stress conditions (Fig. 4). In salt-stressed plants, CAT activity reached 433.74 µmol min^−1^ mg^−1^ FW, a considerable increase compared to the control (214.74 µmol min^−1^ mg^−1^ FW), indicating activation of antioxidative defense mechanisms. All biostimulant treatments further elevated CAT activity relative to the salt-stressed group. The highest values were recorded with PGPR and vermicompost applications, at 1043.05 and 1027.78 µmol min^−1^ mg^−1^ FW, corresponding to increases of 140.5% and 137.0%, respectively. Chitosan (817.33 µmol min^−1^ mg^−1^ FW) and AMF (714.77 µmol min^−1^ mg^−1^ FW) also led to substantial increases of 88.4% and 64.8%, respectively. In contrast, the increases observed with amino acid (543.62 µmol min^−1^ mg^−1^ FW) and fulvic acid (487.29 µmol min^−1^ mg^−1^ FW) applications were more moderate, amounting to 25.3% and 12.3%, respectively.

#### Glutathione reductase (GR)

GR activity was relatively low under control conditions (24.20 µmol min^−1^ mg^−1^ FW) and showed only a slight increase under salt stress, reaching 24.41 µmol min^−1^ mg^−1^ FW representing a negligible change (Fig. 4). Among the biostimulant treatments, vermicompost demonstrated the most pronounced effect, elevating GR activity to 48.22 µmol min^−1^ mg^−1^ FW, corresponding to a 97.5% increase compared to the salt-stressed plants. Amino acid and PGPR applications were similarly practical, enhancing GR levels to 42.29 and 42.34 µmol min^−1^ mg^−1^ FW, respectively, with approximately 73% increases. AMF and chitosan treatments also contributed to improved GR activity, reaching 38.48 µmol min^−1^ mg^−1^ FW (a 57.6% increase) and 33.24 µmol min^−1^ mg^−1^ FW (a 36.2% increase), respectively. In contrast, fulvic acid treatment resulted in a slight decline to 23.10 µmol min^−1^ mg^−1^ FW and did not significantly enhance GR activity under saline conditions.

#### Superoxide dismutase (SOD)

SOD activity increased under salt stress, rising from 400 to 499 U mg^−1^ FW, which corresponds to a 24.8% enhancement compared to the control (Fig. 4). This increase reflects an induced antioxidant defense response. Biostimulant applications further amplified SOD activity, with the most notable effect observed in vermicompost-treated plants, where activity reached 740 U mg^−1^ FW a 48.3% increase relative to the salt-stressed group. PGPR and AMF treatments also provided substantial improvements, raising SOD levels to 654 and 622 U mg^−1^ FW, representing increases of 31.1% and 24.6%, respectively. In contrast, chitosan and fulvic acid resulted in more moderate enhancements of 11.8% and 10.4%, while amino acid treatment had a minimal effect, increasing SOD activity by only 1.4%.

### Lipid peroxidation (measured as MDA content)

**Fig. 5:**
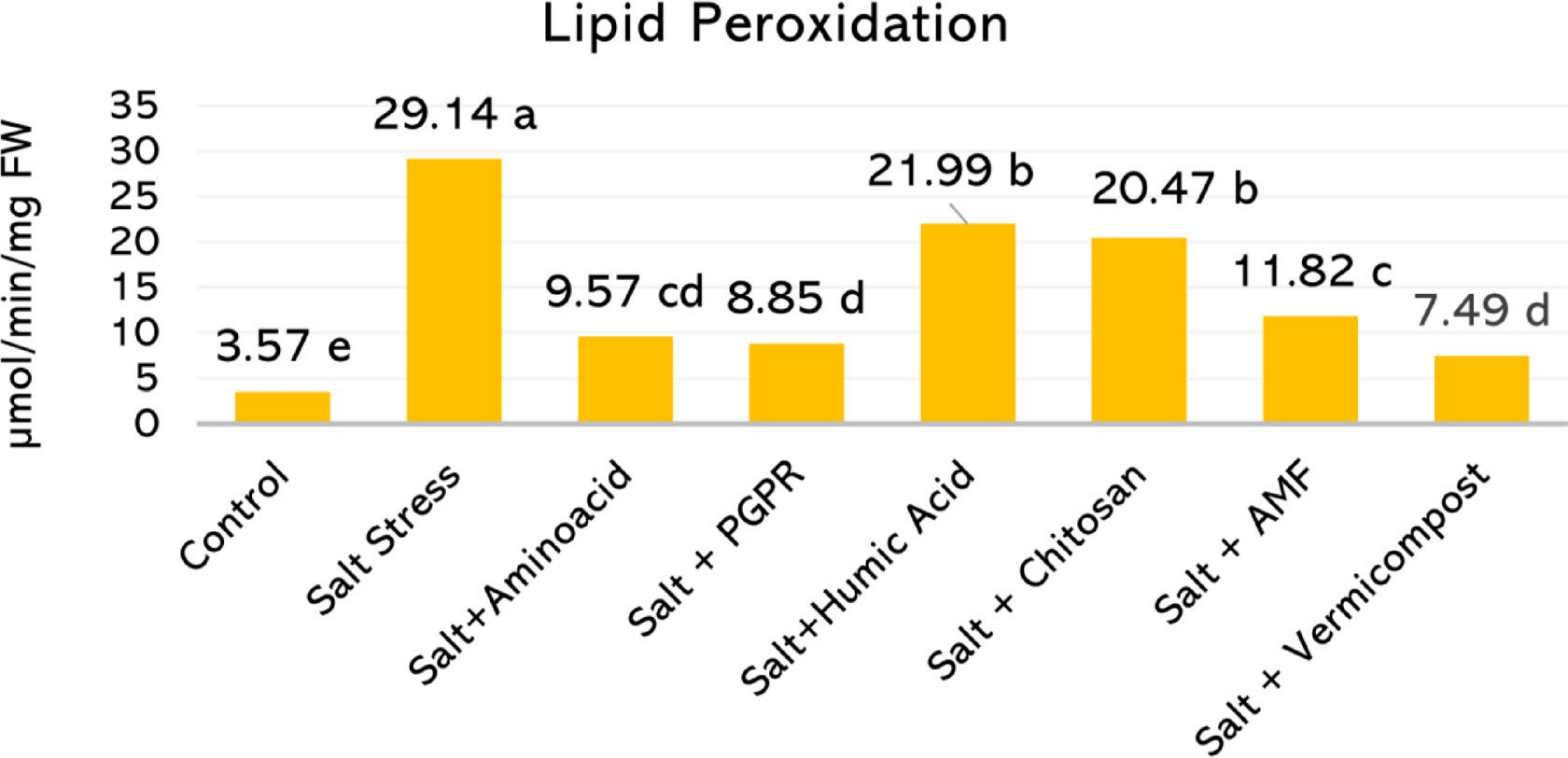
Lipid peroxidation levels in hydroponically grown basil leaves under 50 mM salt stress and biostimulant treatments. Histograms sharing the same letter are not significantly different (*p* < 0.05).

Malondialdehyde (MDA) content, a key indicator of lipid peroxidation and oxidative membrane damage, was substantially elevated under salt stress, reaching 29.14 µmol g^−1^ FW (Fig. 5). In contrast, biostimulant applications effectively mitigated MDA accumulation. The lowest MDA level was observed in the vermicompost treatment (7.49 µmol g^−1^ FW), representing a 74.3% reduction compared to the salt-stressed group. PGPR and amino acid treatments also significantly reduced lipid peroxidation, lowering MDA levels to 8.85 and 9.57 µmol g^−1^ FW, corresponding to decreases of 69.6% and 67.2%, respectively. The AMF treatment led to a 59.4% reduction, with an MDA level of 11.82 µmol g^−1^ FW. In contrast, the treatments of chitosan (20.47 µmol g^−1^ FW) and humic acid (21.99 µmol g^−1^ FW) resulted in more moderate reductions of 29.8% and 24.5%, respectively.

### Proline accumulation

**Fig. 6:**
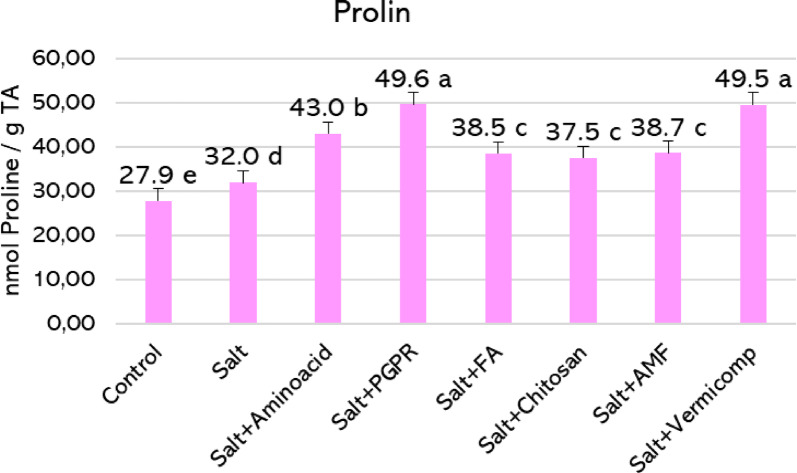
Proline levels in hydroponically grown basil under 50 mM salt stress with biostimulant treatments. Histograms sharing the same letter within the same color group are not significantly different (*p* < 0.05).

Proline levels showed a slight increase under salt stress, rising from 27.90 to 32.00 nmol g^−1^ FW an approximate increase of 15% compared to the control (Fig. 6). Biostimulant applications significantly enhanced proline accumulation beyond the levels observed under salt stress. The PGPR and vermicompost treatments recorded the highest increases, reaching 49.60 and 49.50 nmol g^−1^ FW, corresponding to 55.0% and 54.7% increases, respectively. Amino acid treatment also resulted in a notable elevation (43.00 nmol g^−1^ FW), representing a 34.4% increase. In contrast, humic acid, chitosan, and AMF treatments induced more moderate improvements, ranging from approximately 17.2% to 20.9% compared to the salt-stressed plants.

### Multivariate assessment of biostimulant effects on salt-stressed hydroponic basil

**Fig. 7:**
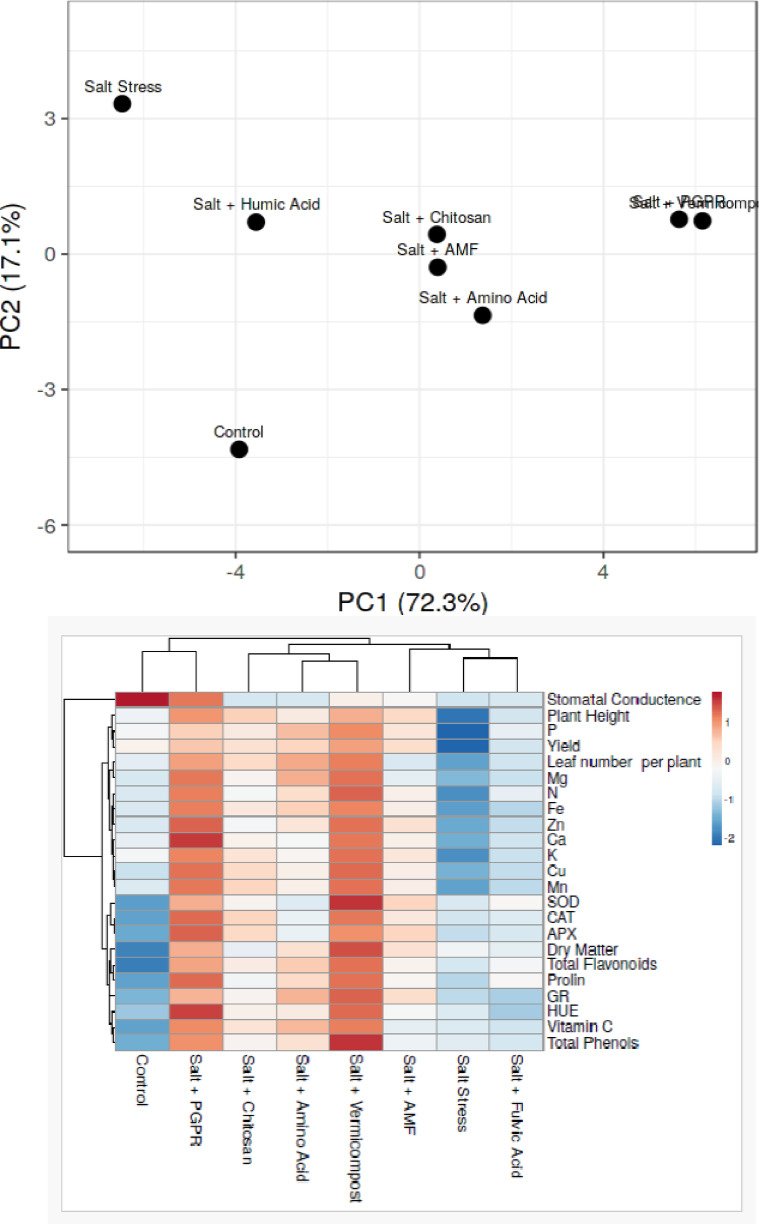
Heat map and PCA plot showing the effects of biostimulant treatments on physiological, biochemical, and nutritional parameters of hydroponically grown basil under salt stress.

Principal component analysis (PCA) was conducted to evaluate the overall response of the treatments based on multiple physiological and biochemical parameters (Fig. 7). The first two principal components, PC1 and PC2, accounted for 72.28% and 17.11% of the total variance, respectively, explaining a cumulative variance of approximately 89.4%. The control and salt-stressed plants were positioned far apart in the PCA plot, indicating a clear response divergence. PGPR and vermicompost treatments clustered closely, suggesting similar and strong alleviative effects under salt stress. Amino acid, AMF, and chitosan treatments were located in intermediate positions, reflecting moderate improvements in plant responses relative to the salt-stressed group.

The heat map summarizes how different biostimulant treatments influenced a wide range of physiological, biochemical, and yield-related parameters in basil under salt stress (Fig. 7). Among the treatments, Salt + Vermicompost and Salt + PGPR exhibited the strongest positive associations (indicated by intense red shading) across most traits, including yield**,** plant height**,** leaf number**,** stomatal conductance**,** antioxidant enzyme activities (SOD, CAT, APX), and quality-related parameters such as vitamin C**,** total phenols**,** and flavonoids**.** While the Salt + Amino Acid treatment also improved several parameters particularly proline**,** GR, and specific biochemical markers it showed slightly more moderate associations than vermicompost and PGPR, suggesting a relatively lower overall impact on plant performance. In contrast, the Salt Stress group (without biostimulants) exhibited strong negative responses across nearly all traits (blue shading), confirming the adverse effects of salinity. The control treatment showed moderate to low values for most parameters, reflecting the absence of stress and the lack of biostimulant-induced enhancement.

## Discussions

Salinity is a major abiotic stress exacerbated by global climate change, particularly affecting hydroponic systems in regions with limited freshwater availability. Basil, a commercially important leafy vegetable after lettuce, is widely cultivated in hydroponics but remains highly sensitive to salt stress. This study aimed to evaluate the potential of selected biostimulants to mitigate salinity-induced damage in basil. Although previous studies have demonstrated the salinity-alleviating effects of biostimulants in various crops^14,59,60^, limited data are available for basil specifically.

Our findings confirmed that salt stress significantly reduced basil growth, yield, and quality by inducing morphological and physiological disturbances. However, biostimulant treatments effectively mitigated these adverse effects, as evidenced by improved plant height, higher dry matter content, and increased total yield. In particular, biostimulant-treated plants under salt stress showed enhanced stomatal conductance, elevated antioxidant activity, increased proline accumulation a key osmoprotectant and reduced membrane damage, as indicated by lower lipid peroxidation. These results are consistent with previous studies reporting similar stress-mitigating effects of biostimulants on various crops^14,61–64^.

### Impact of biostimulant applications on basil growth and yield

It is well known that elevated salinity levels significantly reduce basil yield, with reported losses reaching up to 46% under 60 mM NaCl^63–65^. In line with these findings, our study revealed a 42% decline in basil growth and yield under salt stress. However, the application of biostimulants markedly alleviated these negative effects (Fig. 2). Among the treatments, PGPR and vermicompost were the most effective, increasing yield by 93.3% and 104.2%, respectively, compared to the salt-stressed control. These were followed by amino acids, AMF, and chitosan, which also improved yield substantially. In contrast, fulvic acid showed limited efficacy.

These findings align with previous reports highlighting the beneficial effects of PGPR and AMF on basil productivity under stress conditions^66, 67^. Similarly, biostimulants have been shown to enhance yield in hydroponically grown lettuce exposed to salinity by improving nutrient uptake, stomatal conductance, antioxidant activity, and osmotic regulation^14,47,51,52^. Such physiological improvements not only enhance plant vigor and chlorophyll content but also contribute to increased biomass and yield. Additionally, biostimulants can enhance microbial activity and promote root development, further supporting growth under saline conditions^68,69^.

### Biostimulants enhanced basil color and dry matter

Salt stress stimulated anthocyanin accumulation in purple basil, leading to deeper pigmentation and increased HUE values (Table 1). As HUE is a quantitative indicator of leaf coloration, higher values reflect a shift toward the red-purple spectrum and are often linked to enhanced antioxidant capacity^70,71^. In our study, the application of biostimulants particularly vermicompost and PGPR further increased HUE under salinity, suggesting their role in promoting pigment biosynthesis. This is consistent with earlier reports indicating that salt stress triggers secondary metabolism, including anthocyanin production, as part of the plant’s defense system^72,73^. In line with this, PGPR application has been shown to enhance pigment accumulation in purple basil by modulating hormonal responses and delaying senescence^64,68^.

PGPR application also resulted in the highest dry matter content in basil leaves (11.78%), representing a 20% increase over the control (6.97%) and a notable improvement compared to the salt-stressed plants (9.84%) (Table 1). In aromatic herbs such as basil, dry matter content serves as a key quality parameter, as it reflects the concentration of valuable compounds like essential oils, which contribute to aroma and flavor intensity^74^. Additionally, higher dry matter improves post-harvest shelf life by lowering water content, thereby reducing microbial spoilage and preserving marketability.

### Biostimulants enhance antioxidant accumulation

In our study, salt stress triggered the accumulation of antioxidant compounds in basil leaves, including vitamin C, total phenolics, and flavonoids (Table 2). This response reflects the plant’s adaptive mechanism to oxidative stress. Notably, the application of biostimulants under saline conditions further enhanced antioxidant levels, with vermicompost and PGPR showing the strongest effects. Compared to salt-stressed plants, vermicompost increased total phenolic content by 46.0%, flavonoids by 37.5%, and vitamin C by 20.5%. Similarly, PGPR enhanced total phenolics by 34.0%, flavonoids by 31.3%, and vitamin C by 19.7%. Amino acid treatment also provided considerable improvements, particularly in flavonoid content (+ 25.2%). These findings highlight the role of biostimulants in reinforcing the antioxidant defense system of basil under salinity, potentially supporting better stress tolerance and metabolic balance.

This trend aligns with previous studies showing that salt stress induces phenolic and flavonoid biosynthesis as part of the plant’s protective response^75–77^. Biostimulants can intensify this response by supplying metabolic precursors (e.g., phenylalanine) and modulating defense-related signaling pathways^78,79^. For instance, Ikiz et al.^14^ observed that PGPR significantly increased antioxidant components in hydroponically grown lettuce under salt stress. Moreover, these improvements contribute not only to plant resilience but also enhance the nutritional value of basil, as antioxidants like vitamin C and flavonoids play a vital role in human health. The results support the integration of biostimulants into protected cultivation systems to simultaneously improve crop quality and environmental adaptability, as emphasized by Gruda et al.^80^.

### Effects of biostimulants on nitrate content of basil

Unlike their pronounced effects on antioxidant accumulation, the biostimulants used in our study did not significantly reduce nitrate concentrations in basil leaves under salt stress (Table 2). For instance, the nitrate level in the salt treatment was 180.00 mg kg^−1^ DW, whereas PGPR and vermicompost treatments recorded slightly higher values of 186.53 and 208.90 mg kg^−1^ DW, respectively. This suggests that while biostimulants enhanced nutrient uptake under salinity, they did not promote nitrate assimilation into organic forms.

One possible explanation is that the alleviation of salt-induced stress decreased the plant’s demand for rapid nitrate metabolism, leading to its accumulation. Moreover, the increased availability of nitrogen from biostimulants such as amino acids and vermicompost may have further contributed to elevated nitrate levels. These findings are consistent with previous reports indicating that improved nutrient absorption does not always correlate with reduced nitrate accumulation^47,81^.

### Effects of biostimulants on sodium content of basil

Exposure to salt stress led to a substantial increase in sodium (Na⁺) accumulation in basil leaves. However, biostimulant applications significantly mitigated this effect, as shown in Fig. 3. The greatest reduction in Na⁺ content was observed with vermicompost, which lowered leaf sodium levels by 69% compared to the salt-stressed control. This was followed by amino acid (66%), PGPR (63%), chitosan (56%), AMF (58%), and fulvic acid (36%) treatments. These findings demonstrate the ability of biostimulants to restrict excessive Na⁺ uptake under saline conditions, thereby helping to maintain cellular integrity and ionic homeostasis in basil plants^14^.

### Biostimulants improve nutrient uptake under salinity

Salt stress significantly disrupted the mineral nutrient profile in basil, reducing nitrogen, phosphorus, potassium, calcium, and magnesium levels compared to non-stressed plants (Table 4)^82^. This is likely due to the antagonistic effect of sodium on nutrient uptake. Biostimulant applications alleviated these effects by improving nutrient availability and maintaining ionic balance. Among the treatments, PGPR and vermicompost were particularly effective, increasing most nutrient concentrations by over 140% compared to the salt-stressed control. PGPR likely contributed through the production of organic acids, siderophores, and ACC-deaminase, which improve nutrient solubility and root function^83,84^. The elevated phosphorus content observed in PGPR-treated plants may also reflect the phosphate-solubilizing ability of strains like *Bacillus*, *Rhizobium*, and *Pseudomonas*. Similarly, vermicompost enhanced nutrient uptake, likely due to its rich composition and ability to improve cation exchange capacity^14^. The combined effect of biostimulants in supporting microbial activity, enhancing nutrient solubility, and facilitating ion balance appears to be central to their role in alleviating salinity-induced nutritional imbalances in basil.

### Biostimulant influence on stomatal conductance

Salt stress reduces stomatal conductance by triggering the accumulation of abscisic acid (ABA), which promotes stomatal closure to limit water loss^85^. While this response helps conserve water, it also restricts CO₂ uptake, decreases photosynthesis, and slows plant growth^86,87^. In our study, salt stress significantly decreased stomatal conductance in basil leaves, reducing it to 52.00 mmol m^−2^ s^−1^. Biostimulant applications alleviated this effect, with PGPR showing the strongest response restoring stomatal conductance to 130.00 mmol m^−2^ s^−1^, a 150% increase compared to the salt-stressed control. Vermicompost and AMF also improved conductance by 58.2% and 48.1%, respectively (Table 5)^14^. These improvements align with previous reports suggesting that biostimulants moderate ABA accumulation and promote regulated stomatal behavior, enhancing gas exchange and water use efficiency under stress conditions^85,88^.

### Biostimulants and antioxidant enzyme activity

Salinity stress increases the production of reactive oxygen species (ROS), which cause oxidative damage to cellular structures and impair plant growth^60,78,89^. To minimize these effects, plants activate antioxidant defense systems involving enzymatic and non-enzymatic components. Key antioxidant enzymes such as superoxide dismutase (SOD), catalase (CAT), ascorbate peroxidase (APX), and glutathione reductase (GR) play central roles in ROS detoxification^14^. In our study, biostimulant applications under salt stress significantly enhanced the antioxidant defense system in basil. The activities of SOD, CAT, APX, and GR were notably increased (Fig. 4), indicating improved enzymatic protection. Non-enzymatic antioxidants including vitamin C, phenolics, and flavonoids were also elevated, as shown in Table 2. In addition, lower malondialdehyde (MDA) levels reflected reduced lipid peroxidation, while higher proline accumulation supported osmotic adjustment and stress tolerance^14,48,55,56^.

### Lipid peroxidation and proline accumulation

Lipid peroxidation is a major consequence of oxidative stress, and malondialdehyde (MDA) is widely used as a marker of membrane damage under salinity^60^. In our study, salt stress significantly increased MDA levels in basil leaves. Biostimulant applications reduced this effect, with vermicompost achieving the greatest reduction (74%), followed by PGPR (70%) and AMF (59%). Chitosan and fulvic acid provided more moderate reductions of about 25–30% (Fig. 5). These results indicate that biostimulants help maintain membrane integrity by alleviating oxidative damage caused by salt stress^14^.

Salinity also triggered the accumulation of proline, a key osmoprotectant involved in stress tolerance. Proline functions as both an antioxidant and an ion toxicity buffer, helping to stabilize cellular functions under stress^90,91^. In our study, salt-stressed plants showed a 14% increase in proline compared to the non-stressed control (Fig. 6). Biostimulant treatments further enhanced proline accumulation. PGPR and vermicompost resulted in the highest increases, around 55%, while amino acids led to a 34% rise. Other treatments, including humic acid, chitosan, and AMF, induced moderate increases between 17 and 21%. These findings suggest that specific biostimulants, particularly PGPR, stimulate proline biosynthesis and enhance stress tolerance mechanisms in basil^14^.

### Heat map and PCA analyses

Multivariate analyses further supported the findings of this study. The heat map and principal component analysis (PCA) revealed that vermicompost, PGPR, and amino acid treatments formed distinct clusters associated with improved growth, nutrient uptake, and antioxidant capacity under salt stress. These treatments consistently outperformed others across multiple parameters, indicating their broad-spectrum effectiveness. Chitosan and AMF showed moderate clustering, while fulvic acid grouped closer to the salt-stressed control, suggesting lower efficacy. The use of PCA and heat mapping has also proven effective in previous studies for differentiating treatment responses to biostimulants under salinity stress^92^.

## Conclusion

This study demonstrates that biostimulants offer a promising strategy to counteract the detrimental effects of salinity in soilless basil production. Among the six biostimulants tested, plant growth-promoting rhizobacteria (PGPR) and vermicompost were the most effective in enhancing growth, yield, and quality parameters under 50 mM NaCl stress. These treatments supported antioxidant defense, improved nutrient uptake, reduced oxidative damage, and contributed to osmotic regulation, thereby strengthening basil’s physiological tolerance to salinity. The results highlight the practical potential of PGPR and vermicompost as sustainable tools to improve salt stress resilience in floating culture systems irrigated with marginal water. Future studies should investigate the performance of biostimulants under higher salinity levels and poor-quality irrigation water, as well as their effects on different basil cultivars, particularly focusing on quality-related traits such as essential oil content and aroma profile.

## Data Availability

The data that support the findings of this study are available from the co-corresponding authors upon reasonable request.
